# Multifarious Linkages Between Personality Traits and Psychological Distress During and After COVID-19 Campus Lockdown: A Psychological Network Analysis

**DOI:** 10.3389/fpsyt.2022.816298

**Published:** 2022-06-30

**Authors:** Tzu-Hsuan Liu, Yiwei Xia, Zhihao Ma

**Affiliations:** ^1^School of Political Science and Public Administration, Huaqiao University, Quanzhou, China; ^2^School of Law, Southwestern University of Finance and Economics, Chengdu, China; ^3^Computational Communication Collaboratory, School of Journalism and Communication, Nanjing University, Nanjing, China

**Keywords:** COVID-19, psychological distress, Big Five personality, lockdown, network analysis

## Abstract

**Background:**

The novel coronavirus disease pandemic is still proliferating and is not expected to end any time soon. Several lockdowns and social distancing measures might be implemented in the future. A growing body of research has explored the effect of personality on individuals' psychological wellbeing during the pandemic. However, most prior studies have not discussed the dynamic and reciprocal transactions between personality and psychological distress in various situations. Therefore, this study aims to explore the internal mechanisms of the ways in which certain personality traits triggered specific symptoms during and after college lockdown, by using network analysis.

**Methods:**

Based on survey data from 525 university students in China, the study detected the connection between individual personality and psychological distress through network analysis. Of the participants, 70.1% were female, and 20.9% were male. The mean age of the participants was 19.701 (SD = 1.319) years. We estimated networks *via* two steps: First, two networks that only contain the Big Five personality traits and the six symptoms of psychological distress during and after the lockdown measure were estimated. Second, we add control variables and re-estimated the networks to check whether the linkages among the Big Five personality traits and the six symptoms of psychological distress observed in the first step were stable. Moreover, we employed strength centrality as the key indicator to present the potential significance of diverse variables within a network.

**Results:**

The findings demonstrate that, first, “depress” was the central symptom in the network during the college lockdown, while “efforts” was the central symptom after the lockdown. Second, the symptoms of “restless” and “worthless” significantly declined after the lockdown. Third, we found that there is an internal mechanism through which personality affected certain psychological symptoms during and after lockdowns. Specifically, neuroticism triggered certain symptoms during and after the lockdown, while extraversion and conscientiousness suppressed certain symptoms. Substantial evidence on internal linkages is imperative to develop effective interventions.

**Conclusion:**

This study explores the internal mechanisms of the ways in which certain personality traits trigger specific symptoms. Overall, our results provide empirical evidence that personality traits play a key role in how individuals with certain traits respond to college lockdown during a pandemic. The study makes a significant contribution to the literature because it is among the first few studies which explores the effects of personality traits on individual psychological distress using network analysis during the pandemic.

## Introduction

The novel coronavirus disease (COVID-19) pandemic significantly influenced peoples' daily lives and wellbeing, including physical and psychological wellbeing. For example, research showed that individuals reported more alcohol consumption and poor sleep quality as the pandemic progressed, especially for alcohol consumption and sleep quality (1). Other research found that the pandemic might deteriorate psychological health and exacerbate suicide risk (2, 3). Certain strains of the SARS-CoV-2 virus may continue circulating in pockets of the world population in the future (4). Implementing control measures—such as lockdowns and social distancing—is imperative for controlling the spread of the virus (5). Since the outbreak of COVID-19 in 2019, it has affected university students globally (6); numerous universities closed their campuses and adopted remote teaching *via* online platforms (7). The COVID-19 pandemic has substantially influenced daily lives and wellbeing of college students. For example, a study showed that problematic Internet use (PIU) has become a serious issue among residential college students (8). Another study demonstrated that college students' externalizing problems and attention problems increased after the outbreak of COVID (9). Research related to individual psychological wellbeing found that college students experienced increased acute stress, anxiety, and depressive symptoms during the epidemic (10).

Universities in China employed an innovative closed or semi-closed campus management measure, which derived substantial epidemiological benefits to reduce viral transmission. Almost all universities in China placed teachers and students under blanket campus lockdowns. It is worth noting that not all universities in China implement closed management measure at the same time. It depends on the severity of epidemic and provisional epidemic control policy. Universities in the area with a small outbreak usually adopt closed campus management measure in China. Although students and teachers can return to the campus, some of universities ask teachers to adopt remote teaching to reduce contact. Also, university students are not allowed to leave the campus unless necessary, and off-campus personnel cannot enter the campus without administrative permission. A lack of social interaction, reduced teacher–student contact, and difficult dorm situations that were unfit for learning purposes, involving insufficient data bandwidth, and limited space, directly challenge every student. Although strict precaution management at universities helps to halt the spread of the virus, prior research that connecting lockdowns to subjective wellbeing suggest that strict measures may negatively affect students' psychological wellbeing (11).

For example, Deng et al. examined the prevalence of psychological disorders in students during the COVID-19 pandemic and found that the pooled prevalence of depressive symptoms, anxiety symptoms, and sleep disturbances was 34, 32, and 33%, respectively (12). By conducting meta-analysis. The researchers observed that the symptoms might resulted from a combination of disrupted academic routines, and isolation of university students. Moreover, Allé and Berntsen has found that psychotic symptoms were higher in individuals with less social interactions, more prolonged self-isolation and smaller living space (13). The study underscored the negative effect of social isolation and living in small spaces on psychological distress during the epidemic. Additionally, Benke et al. suggested that a higher level of restrictions due to lockdown measures, a stronger reduction of social contact, and stronger perceived changes in life were linked to poorer psychological health (14). Furthermore, Brooks et al. highlighted that lengthy lockdown aggravates mental health-related issues (15). Similar results have been reported in several other research (16). These findings underlined that COVID-19 lockdowns were associated with higher levels of psychological symptomatology. Although a growing body of research has explored individuals' psychological wellbeing during the COVID-19 lockdowns, only a few studies discuss the changes in individuals' psychological wellbeing during and after the lockdowns. This paper will elucidate the differences in the effect that individuals' internal mechanisms have on their psychological symptomatology, during and after the lockdown.

A fair body of research has highlighted that the five broad personality traits (Big Five model)—extroversion, neuroticism, conscientiousness, agreeableness, and openness (17)—as the key factors affecting psychological distress. Every trait is made up of multiple facets. For instance, extraversion may reflect an approach temperament and positive emotionality. Neuroticism is the trait disposition to experience moodiness, anxiety, and depression. Agreeableness is the trait disposition to be affable, kind, empathic. Conscientiousness reflects qualities of impulse control and reliability. Openness involves several facets, including curiosity, flexibility, imagination, and willingness to devote oneself in unconventional experiences (17). Research has demonstrated that personality traits were differentially associated with psychopathology and positive mental health outcomes. For instance, emotional stability (reversed neuroticism) was associated with psychopathology, while extraversion and agreeableness were associated with positive mental health outcomes (18). A few recent studies exploring the effect of personality traits on psychological distress during the COVID-19 pandemic found that certain personality traits are associated with psychological distress. However, the results were inconsistent. For instance, Kroencke et al. conducted a large-scale experience-sampling study and demonstrated that neuroticism had a negative impact on individuals' mental wellbeing during the pandemic (19). Similar findings have been observed in another research (20). Additionally, negative emotional responses may be driven by extraversion because highly extraverted individuals may be significantly dependent on pandemic information, which in turn increases anxiety related to the pandemic (21). A study found that individuals who were introverted experienced less loneliness during the lockdown in France (22). In contrast, Nikčević et al. observed that extraversion and conscientiousness positively influenced psychological wellbeing during the pandemic (23). The inconsistent findings may be due to two reasons: first, such studies did not take different situations into account (e.g., college lockdown or quarantine) (24); second, they did not examine the effect of personality traits on certain symptoms of psychological distress. The reason that the present study explored individual symptoms rather than the whole picture of wellbeing or distress lies in the fact that psychiatric symptoms have been argued to have internal relationships between each other rather than being effects of common association (25). Borsboom suggested the interactions between psychiatric symptoms can be viewed as a network, in which they are nodes and internal interactions between them are linkages between nodes (25, 26). This network approach can be applied to explore the effect of personality trait on certain symptoms of psychological distress.

Also, according to the transactional stress moderation model, personality may influence physiological and psychological responses in response to stressful circumstances, which may in turn lead to damaging physiological, behavioral, and psychological consequences (27). The transactional views of personality show that personality traits, social environments, and symptoms of psychological distress are not always static and affect each other. Based on this theory, distinct personality traits activate or suppress certain psychological symptoms of psychological distress in various situations.

Although some scholars suggested that the end of the COVID-19 pandemic is near, however, COVID-19 may be recurrent disease that the world has to deal with (28). Therefore, recurrent implementation of lockdowns and social distancing measures may be inevitable. Given that behavioral containment and control measures may negatively affect individual psychological wellbeing, it is important to investigate the internal mechanisms through which personality influences individuals' psychological distress in different situations.

A large body of literature explores the effect of personality traits on psychological distress during the pandemic. However, most studies have not considered the possibility of changes in internal linkages between personality traits and psychological wellbeing under different situations. Furthermore, traditional analysis provides limited exploration of the internal linkages (29). Our study fills this gap by using network analysis, that can provide insights into the effects of personality and psychological distress in various environmental contexts.

Network analysis can provide useful information by graphically mapping the connections between personality and psychological distress; thus, it is possible to assess the specifics of the distress triggered by a certain personality trait in a stressful event (25). Moreover, network analysis helps identify the central symptom and the central trait, which are important predictors of related comorbidities and associations (30, 31). Furthermore, several studies have adopted network analysis to comprehensively disentangle the associations between multiple forms of exposure, such as exposure to traumatic events, and psychological symptoms (32, 33). In addition, network analysis bring benefits to capture nuanced changes of psychological dynamic (34).

Detecting the connection between individual personality and psychological distress through network analysis will helps scholars develop treatment strategies that would most effectively promote enhanced wellbeing in individuals. Although previous research has investigated the impact of personality on psychological distress during the pandemic, the specific triggering effects that personality traits may have on psychological distress remain unexplored. Specifically, whether the mechanism changes after the college lockdown remains unknown.

To identify and support individuals with psychological distress, we emphasized the role of personality traits during and after the closed campus management measure at a university in China. This study aims to explore how the Big Five personality traits influence psychological distress in various environmental contexts.

We explored whether and how this change in responses was affected by personality traits. The Big Five personality traits were chosen to determine internal linkages among personality traits and psychological distress.

## Materials and Methods

### Participants and Procedure

The participants were college students recruited from a large university in China. A total of 546 undergraduate students from one college were surveyed. An incentive was offered for participation in the survey in the form of snacks under 1 USD. The survey was done *via* one Chinese online survey system—wenjuanwang (wenjuan.com). The survey was conducted from October 8 to October 30, 2020, while the closed management of the university was implemented from July 1 to October 1, 2020. It is worth noting that some of universities in China implement closed management measure since summer vacation because there are still a great number of students staying at campus. At the time of the survey, the participants were not precisely aware of when the closed management measure would be implemented again; therefore, the current survey can be considered an effective representation of the potential effects of lockdown on individuals' psychological coping mechanisms.

The survey was conducted on the Big Five personality traits; the participants also rated their levels of psychological distress during and after the implementation of the college lockdown. Twenty-one individuals who refused to participate or did not complete the questionnaire were excluded from the analysis. Finally, 525 individuals were included in the final analysis.

Written informed consent was obtained from all the participants prior to the survey. This study was approved by the research ethics committee of the authors' affiliations.

### Measure

#### Big Five Personality Traits

The Big Five Inventory-10 (BFI-10) was employed to measure participants' extraversion, agreeableness, conscientiousness, neuroticism, and openness (35). The BFI-10 has 10 items (two for each trait) with response options on a 5-point Likert scale, where 1 = strongly disagree and 5 = strongly agree. It was identified as a valid inventory to measure personality traits in culturally diverse populations, including the Chinese population (36). The mean scores of two items for each trait, where reversed items were treated in reverse, were used to present individuals' Big Five personality traits.

#### Psychological Distress During and After College Lockdown

The Kessler Psychological Distress Short Scale (K6) was used to identify participants' psychological distress (37). The K6 scale consists of six questions regarding feeling “nervous,” “hopeless,” “restless or fidgety,” “so depressed that nothing could cheer you up,” “that everything is an effort,” and “worthless” during a certain period. Each item presents a psychological distress symptom (Table 1).

**Table 1:** K6 items and corresponding reference names.

**Reference names**	**Items**
Nervous	During/After college lockdown, about how often did you feel nervous?
Hopeless	During/After college lockdown, about how often did you feel hopeless?
Restless	During/After college lockdown, about how often did you feel restless or fidgety?
Depress	During/After college lockdown, about how often did you feel so depressed that nothing could cheer you up?
Effort	During/After college lockdown, about how often did you feel that everything was an effort?
Worthless	During/After college lockdown, about how often did you feel worthless?

The measurements of psychological distress were dependent on the self-report retrospective questionnaire. Following Swedo et al. all participants were asked to rate their psychological distress in two different periods: during the college lockdown and after the lockdown (38). The response options were on a seven-point scale (where 1 = none of the time and 7 = all the time). The mean score of the six items was used to measure psychological distress, with higher scores indicating more severe psychological distress. The K6 scale used in the current study presents excellent reliability (Cronbach's alpha = 0.908 for psychological distress during the lockdown, Cronbach's alpha = 0.937 for psychological distress after lockdown).

#### Control Variables

Sex, age, and grade were used as control variables in the current study. Sex was coded as female or male, age was presented by year, and the grade was separated as Grade 1, Grade 2, Grade 3, and Grade 4 according to the university students' information system.

### Statistical Analysis

#### Descriptive Statistics

A descriptive analysis was first applied to provide an outline of the sample. Second, a descriptive comparison of psychological distress, including six symptoms, during and after the university lockdown measure, was conducted to observe potential variations in psychological distress in different periods. The paired *t*-test was used for statistical comparison. Third, we conducted the correlation test to reveal the relationships among personality traits and psychological distress during and after college lockdown.

#### Network Analysis

In this study, we estimated undirected networks in the form of a mixed graphic model, in which the Big Five personality traits, six symptoms measured by the K6, and control variables were treated as nodes, and edges among nodes can be interpreted as partial correlation coefficients among these variables (39). Since many control variables, such as sex and grade, were categorical, we estimated networks *via* the R package mgm, which was designed to perform network analysis with diverse types of variables (e.g., binary, categorical, and counts) rather than continuous only (40).

Because the network analysis with control variables could make the visualization more complicated, we estimated networks *via* two steps: First, two networks that only contain the Big Five personality traits and the six symptoms of psychological distress during and after the lockdown measure were estimated. Second, we add control variables and re-estimated the networks to check whether the linkages among the Big Five personality traits and the six symptoms of psychological distress observed in the first step were stable. Given that mgm provides the algorithm of regularized generalized regression, we adopted the extended Bayesian information criterion (EBIC) with tuning parameter γ = 0.5, as suggested by a previous simulation study (41).

#### Centrality Analysis

We employed strength centrality as the key indicator to present the potential significance of diverse variables within a network (39). Strength centrality is defined as the sum of all the absolute weights of the directly connected edges of a node. Given that the centrality indicators of nodes may be significantly influenced by control variables that are usually highly correlated, the interpretation of nodes' significances within a certain network should exclude those control variables.

#### Robustness Analysis

To assess the accuracy and stability of the edges and the strength centrality obtained from the network analysis, we employed the following steps to perform the robustness analysis. First, we use the bootstrapping approach to estimate the 95% confidence interval to determine the accuracy of the edges. Second, the centrality-stability (CS) coefficient was used to assess the stability of node strength centrality. As suggested by Epskamp et al., the CS coefficient should be above 0.25, revealing adequate stability, and a CS coefficient higher than 0.5 reveals preferable stability (39). We conducted a robustness analysis using the R package bootnet, and all models were bootstrapped 1,000 times.

## Results

### Descriptive Statistics

Table 2 presents the results of descriptive statistics. The sample consisted of 525 college students with a mean age of 19.701 years. Of the participants, 70.1% were female, and the distribution of grade diversity was relatively balanced; the percentage of each grade among the total participants ranged from 20.8 to 27.6%. Both the scores of psychological distress during and after the lockdown measure were at a relatively low level, and the mean score of K6 after lockdown (M = 2.461, SD = 1.307) was slightly lower than the mean score of K6 during the lockdown (M = 2.509, SD = 1.241). Additionally, participants reported moderate levels in the five domains of personality traits.

**Table 2:** Descriptive statistics (*N* = 525).

	**Mean (SD)**	***N* (%)**	**Min**	**Max**
**Big five personality traits**				
Extraversion	2.818 (0.887)	-	1	5
Agreeableness	3.509 (0.749)	-	1	5
Conscientiousness	2.751 (0.790)	-	1	5
Neuroticism	3.153 (0.789)	-	1	5
Openness	3.571 (0.855)	-	1	5
**Mental illness**				
K6 score during lockdown^a^	2.509 (1.241)	-	1	7
K6 score after lockdown^a^	2.461 (1.307)	-	1	7
**Control variables**				
Sex (1 = female)	-	368 (70.1%)	0	1
Age (years)	19.701 (1.319)	-	15	25
*Grade (ref. = Grade 1)*	-	-	-	-
Grade 2	-	145 (27.6%)	0	1
Grade 3	-	128 (24.4%)	0	1
Grade 4	-	109 (20.8%)	0	1

a *The score refers to the average value of six items*.

Table 3 shows the descriptive comparison of the K6 score and the scores of the six symptoms during and after the lockdown measure. The mean difference of K6 scores between during and after the lockdown measure was not statistically significant (Diff. = 0.048, T = 1.486), while two of the six symptoms, namely “restless” (Diff. = 0.137, T = 2.715) and “worthless” (Diff. = 0.166, T = 3.495) present a significant decrease after the lockdown measure.

**Table 3:** Mean differences of K6 score and six symptoms' scores between during and after lockdown (*N* = 525).

	**During lockdown**	**After lockdown**	**Differences**	***T***
K6 score^a^	2.509 (1.241)	2.461 (1.307)	0.048	1.486
Nervous	2.701 (1.445)	2.750 (1.489)	−0.050	−0.979
Hopeless	2.008 (1.286)	2.057 (1.377)	−0.050	−1.130
Restless	2.954 (1.626)	2.817 (1.595)	0.137	2.715^**^
Depress	2.484 (1.478)	2.423 (1.508)	0.061	1.280
Effort	2.510 (1.498)	2.488 (1.477)	0.023	0.447
Worthless	2.396 (1.635)	2.230 (1.544)	0.166	3.495^***^

a *The score refers to the average value of six items*.

** *p < 0.01*,

*** *p < 0.001*.

**Figure 1:**
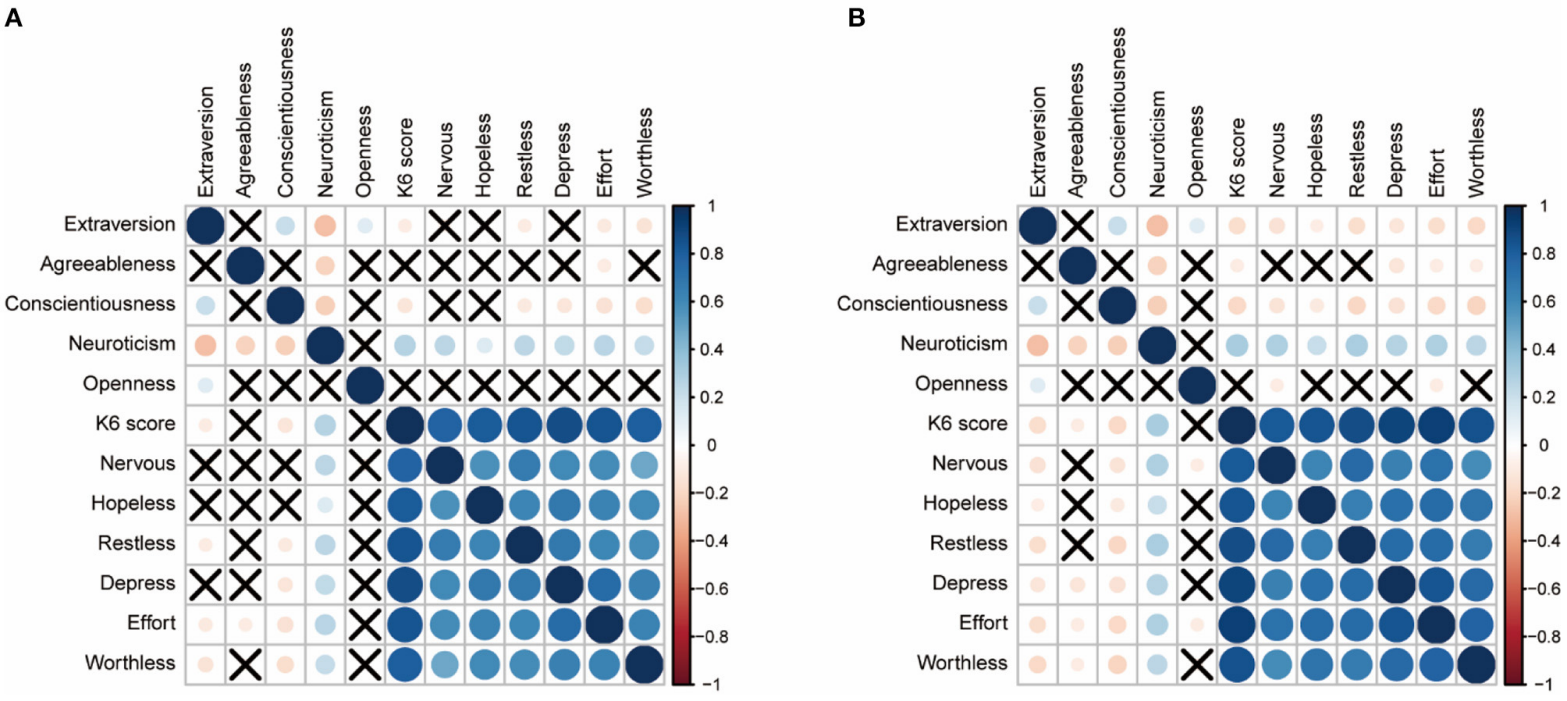
Correlation results of the relationships among Big Five personality traits, K6 mean score, and all symptoms. Crosses in cells denote insignificant correlations. **(A)** Correlation matrix during college lockdown. **(B)** Correlation matrix after college lockdown.

Figure 1 shows the correlation results of the relationships among Big Five Personality Traits, K6 mean score, and all symptoms during and after college lockdown. Detailed correlation coefficients were listed in Supplementary Tables 1, 2.

Both Figures 1A,B reveal that neuroticism was negatively correlated with extraversion, agreeableness, and conscientiousness. Extraversion was positively correlated with conscientiousness and openness.

Neuroticism was positively correlated with the K6 mean score and six psychological distress symptoms during and after college lockdown. However, the other four traits' associations with K6 means score and six psychological distress symptoms varied. During college lockdown, extraversion was negatively correlated with K6 mean score and three symptoms (“restless,” “effort,” and “worthless”), conscientiousness was negatively correlated with K6 mean score and four symptoms (“restless,” “depress,” “effort,” and “worthless”), agreeableness only negatively correlated with one symptom (“effort”), openness was insignificantly correlated with any psychological distress symptom and the K6 mean score. After the college lockdown, both extraversion and conscientiousness were negatively correlated with K6 mean score and all psychological distress symptoms, agreeableness was negatively correlated with K6 mean score and three symptoms (“depress,” “effort,” and “worthless”), openness was negatively correlated with two symptoms (“nervous” and “effort”). These results implied that the protective roles of four personality traits may be inhibited during the lockdown period.

### Results of Network Analysis

#### The Structure of Networks During Lockdown

**Figure 2:**
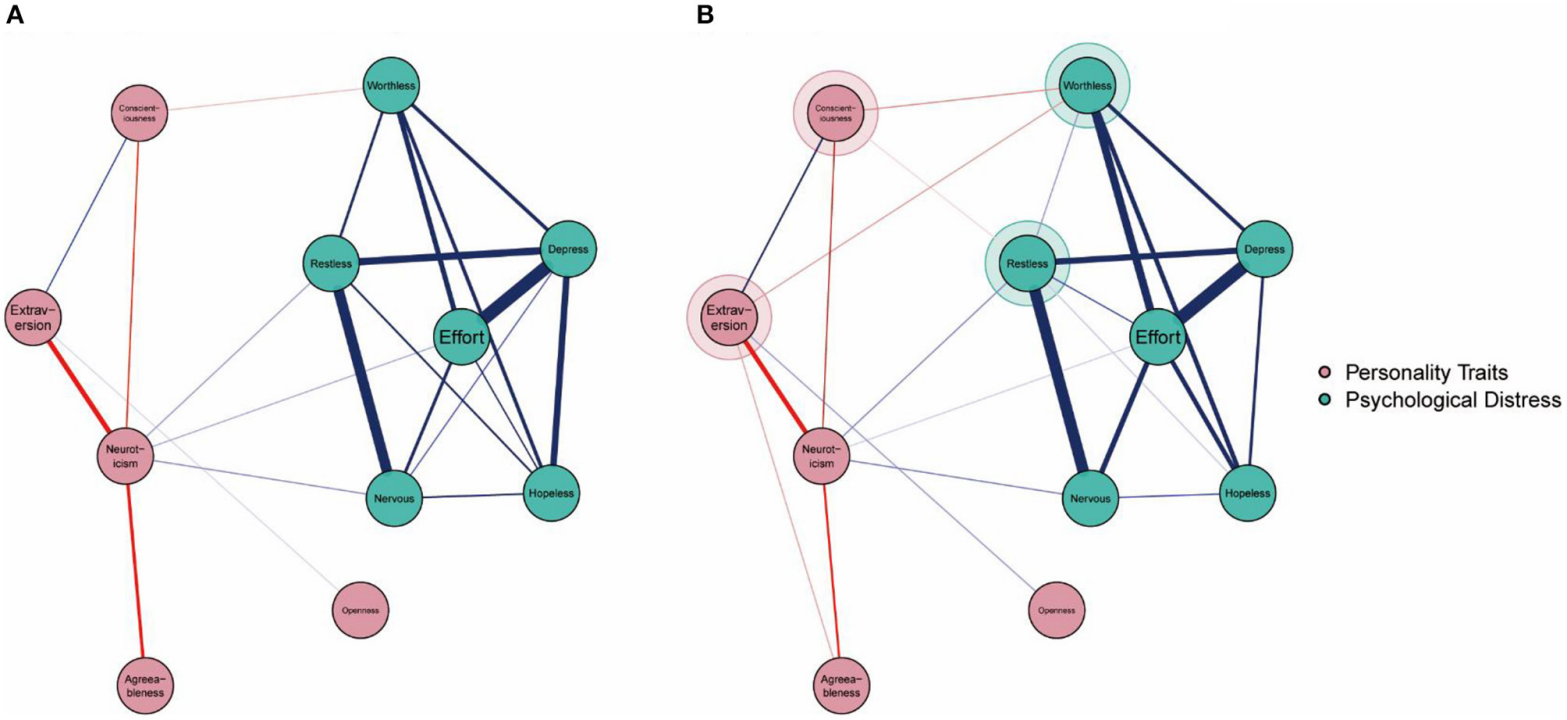
Graphical results of the estimated network models without control variables during college lockdown and after college lockdown. Blue links denote positive associations and red links denote negative associations. The color borders highlighted for nodes in **(B)** denote new links compared to the **(A)**.

**Figure 3:**
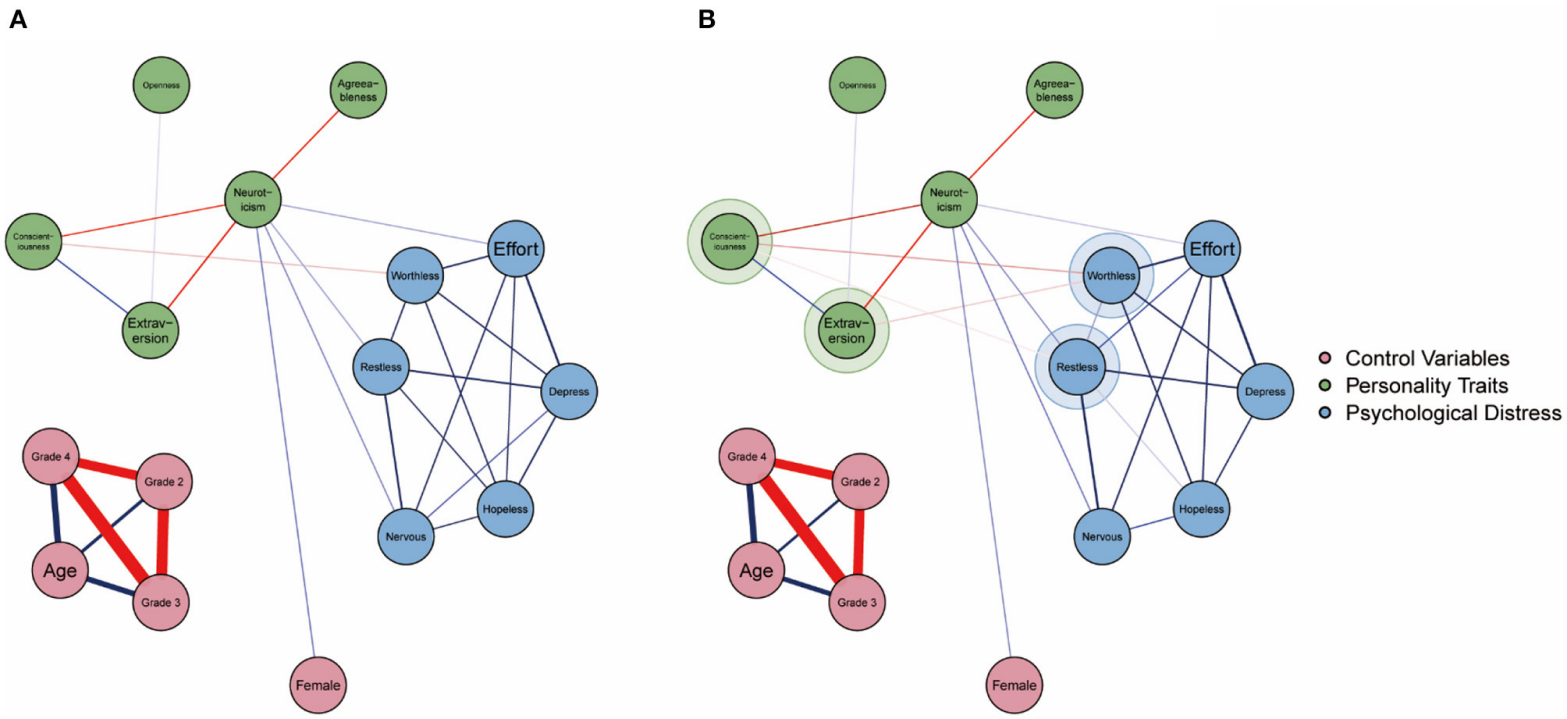
Graphical results of the estimated network models with control variables during college lockdown and after college lockdown. Blue links denote positive associations and red links denote negative associations. The color borders highlighted for nodes in **(B)** denote new links compared to the **(A)**.

The estimated networks of the psychological interaction linkages during lockdown are displayed in Figures 2A, 3A. Detailed edge weights are listed in Supplementary Tables 3, 5, and the bootstrapped accuracy plots are listed in Supplementary Figures 1, 3.

The results in Figure 2A show that all six symptoms of psychological distress were significantly intercorrelated. Moreover, neuroticism was directly positively linked to three symptoms (“nervous,” “restless,” and “efforts”). Additionally, conscientiousness was directly negatively linked to one symptom (“worthless”). The results in Figure 3A show that after controlling for age, sex, and grade, the inter-community connections between the Big Five personality traits and psychological distress symptoms were consistent with the results in Figure 2A.

#### The Structure of Networks After Lockdown

The estimated networks of the psychological interaction linkages after lockdown are displayed in Figures 2B, 3B. Detailed edge weights are listed in Supplementary Tables 4, 6, and bootstrapped accuracy plots are listed in Supplementary Figures 2, 4.

The results in Figures 2B, 3B present more sophisticated patterns compared with the results in Figures 2A, 3A. First, inter-community connections between the Big Five personality traits and psychological distress symptoms revealed in Figures 2A, 3A were stable and consistent with results in Figures 2B, 3B. Second, two new inter-community connections were highlighted: the negative linkages between extraversion and “worthless” and conscientiousness and “restless.”

### Centrality Analysis

The robustness of the strength centrality revealed that the networks were very stable (all CS-coefficients were 0.75), and the stability of the network structures *via* the bootstrapping approach is displayed in Supplementary Figure 5.

**Figure 4:**
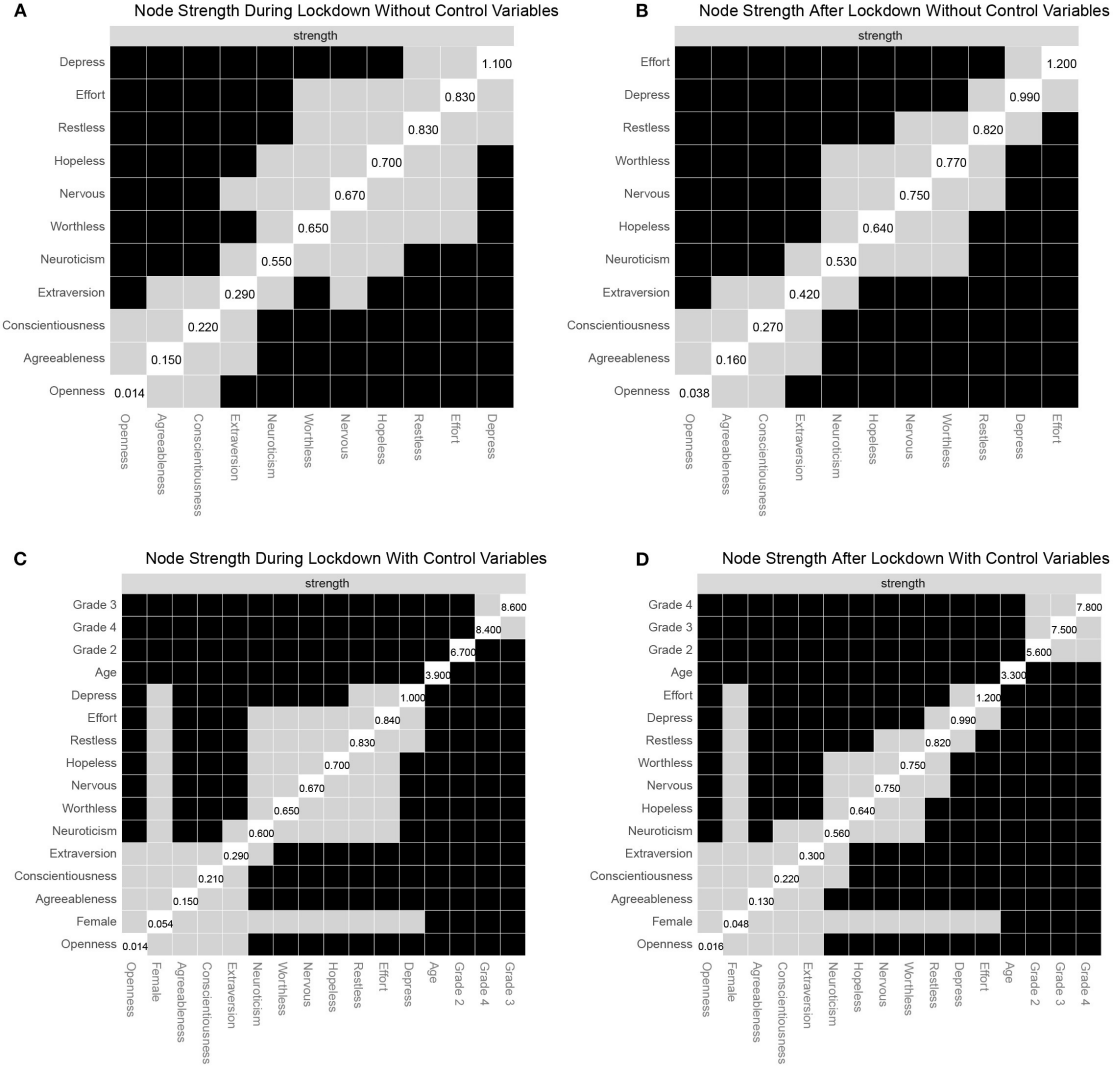
Strength results and non-parametric bootstrapped difference test for strength centrality. Values reported in the diagonal represent the values of strength centrality for each node. Gray boxes denote no difference between nodes, while black boxes indicated significant difference. **(A)** Node strength during lockdown without control variables. **(B)** Node strength after lockdown without variables. **(C)** Node strength during lockdown with control variables. **(D)** Node strength after lockdown without variables.

Figure 4A shows that “depress” was the most central symptom with a significantly larger strength centrality than other symptoms in the network during the lockdown. Moreover, neuroticism was the most central trait and had a significantly higher strength centrality than other personality traits. After excluding the estimated values of control variables, results in Figure 4C were consistent with the above results in Figure 4A.

Figure 4B shows that “efforts” was the most central symptom and had a significantly larger strength centrality than other symptoms in the network during the lockdown. Neuroticism is the most central trait. The results in Figure 4D reveal a consistent pattern after controlling for age, sex, and grade.

## Discussion

This study primarily aims to provide solid empirical evidence to resolve whether and how the Big Five personality traits predict psychological distress and whether the mechanism varies during and after lockdowns. Network analysis offers us valuable insights to develop substantial strategies in response to psychological health crises, by examining the pattern of routine activation of personality on psychological distress in high-stakes contexts. To better understand the relationship between personality and psychological health during and after the COVID-19 lockdowns, we focus on which personality traits trigger certain categories of psychological distress during and after the college lockdown periods. Our study is among the first few studies which explores the effects of personality traits on individual psychological distress using network analysis during the pandemic. We have highlighted several significant findings.

Initially, regarding psychological distress, our findings suggested that “depress” was the most central symptom in the network during the college lockdown. This suggests that lockdown adversely might influence the mental health of the population, which is consistent with previous research reporting an increase in the occurrence and frequency depressive symptoms during the lockdown (42). It is noteworthy that “efforts” was the central symptom after the college lockdown. A possible reason is that individuals understand that COVID-19 may remain in circulation in pockets of world population in the future, which perpetuates their sense of danger and uncertainty. Thus, even if they could leave the campus, they still related to “everything being an effort.” Another possible reason may be that the university still has a strict access control system. For example, students should provide health codes to enter the campus. They must also obtain permission from the university to leave the province.

Also, the symptoms of “restless” and “worthless” experienced a significant decline after the lockdown. The results suggest that lockdown measures might negatively affect the mental health of the population, consistent with previous research reporting an increase in symptoms such as “restless” and “worthless” during the lockdown (43). Lockdowns might be an unsettling experience because of the students' loss of the freedom to go out, the lack of contact with loved ones in person, feelings of boredom, and uncertainty about the future. Lifting lockdown restrictions might relieve the symptoms of “restless” and “worthless”.

Moreover, personality traits play a crucial role in the ways in which individuals with certain traits respond to lockdown measures. In fact, previous research has found that extraversion, neuroticism, and conscientiousness are key predictors of psychological wellbeing (19). In the current study, we found that extraversion, neuroticism, and conscientiousness might be responsible for the activation or suppression of some psychological symptoms during and after lockdowns. First, the findings demonstrated that neuroticism was the most central personality trait during and after college lockdown. During and after the lockdown, neuroticism might activate the symptoms of “nervous,” “restless,” and “efforts.” These findings are consistent with previous evidence, which showed that individuals with higher levels of neuroticism did not adapt well to lockdown, which in turn, might lead to negative impacts on psychological distress during the pandemic (19). Second, conscientiousness might suppress the symptom pertaining to feeling “worthless” during the lockdown, while after the lockdown, it might suppress both symptoms of “worthless” and “restless.” This result is consistent with another study, which suggested that individuals with higher levels of conscientiousness have fewer negative impacts on wellbeing (23). Meanwhile, the symptom of “worthless” might also be suppressed by extraversion after the lockdown. During the lockdown, extraversion might not prevent psychological distress. A possible reason may lie in the fact that individuals high in extraversion may exhibit increased dependence on pandemic information, such as medical information, which in turn facilitates feelings of threat and stress (20). However, after the lockdown, extraverted individuals might have increased contact with other people, which in turn might increase social support. Increase in social support can relieve the symptom of “worthless” (21). These findings are consistent with the transactional model of stress (27). The theory suggests that personality and symptoms of psychological distress are not always static. There might be reciprocal relationships between personality, psychological health, and situational factors (25). The theory supported our findings, which showed that distinct personality traits activate or suppressed certain psychological symptoms.

As for implication, the study suggests that authorities can carry out specific approaches depending on various psychological distress, personality traits, and situations. For instance, neuroticism is the most central symptom during and after lockdowns, and it might trigger symptoms of nervousness, restlessness, and effort. Therefore, universities should target students with high levels of neuroticism and develop targeted interventions, such as mentoring, psychological counseling, and teaching coping skills. Additionally, according to Vos et al.'s study (44), positive personality traits (e.g., optimism, mindfulness, and resilience) the relationship between fear of COVID-19 and psychological distress (45). It is, to our best knowledge, the first study exploring the moderated effect of positive personality traits on the relationship between fear of COVID-19 and psychological distress. Based on the findings of the study, we suggested that the authority can make interventions aimed at improving positive constructs such as optimism, mindfulness, and resilience during the pandemic to promote college students' psychological health.

## Conclusion

This study explores the internal mechanisms of the ways in which certain personality traits trigger specific symptoms. Overall, our results provide empirical evidence that personality traits play a key role in how individuals with certain traits respond to college lockdown during a pandemic. Specifically, neuroticism triggered psychological distress during and after the lockdown, while extraversion and conscientiousness suppressed psychological distress. A comprehensive understanding of the internal linkages between personality and psychological distress is imperative for developing effective interventions. Furthermore, the current study contributes to the literature by using network analysis and elucidates the effects of personality traits on psychological distress.

Although the study made substantial contributions to the existing literature, two limitations of the study need to be considered. First, the cross-sectional nature of the research does not allow us to generalize the causal relationship between the variables. Moreover, the measurements of psychological distress were dependent on the self-report retrospective questionnaire, which may have resulted in retrospective recall biases. However, according to Middel et al.'s study, retrospective measurement of change in a 12-week interval was not significantly linked to recall bias. The present study was conducted right after the school lockdown (46). It had participants reflect on their previous psychological state during and after the lockdown. The time span was within 12-week intervals, which was not so long that they have serious difficulty recalling how they were during the lockdown. Furthermore, the data of this study were collected from one university in China. Closed-campus management in China is innovative and differs from other universities in other countries. Therefore, it may be difficult to generalize the findings universally. In the future, longitudinal data from multiple universities worldwide should be collected to overcome these limitations.

## Data Availability Statement

The raw data supporting the conclusions of this article will be made available by the authors, without undue reservation.

## Ethics Statement

The studies involving human participants were reviewed and approved by the Research Ethics Committee of School of Political Science and Public Administration at Huaqiao University. The patients/participants provided their written informed consent to participate in this study.

## Author Contributions

T-HL wrote the first draft of the study. YX revised the first draft. ZM made the conceptualization and design. All authors approved the final manuscript.

## Funding

This study was supported by Major Project of the National Social Science Fund of China (Grant No. 19ZDA324), Project of Social Science Foundation of Jiangsu Province (Grant No. 21XWC010), the Fundamental Research Funds for the Central Universities (Grant No. 011014370119), and Huaqiao University Start-up Research Fund for High-Quality Professionals (Grant No. 19SKBS214).

## Conflict of Interest

The authors declare that the research was conducted in the absence of any commercial or financial relationships that could be construed as a potential conflict of interest.

## Publisher's Note

All claims expressed in this article are solely those of the authors and do not necessarily represent those of their affiliated organizations, or those of the publisher, the editors and the reviewers. Any product that may be evaluated in this article, or claim that may be made by its manufacturer, is not guaranteed or endorsed by the publisher.
