# Supplementary material for: Multifarious Linkages Between Personality Traits and Psychological Distress During and After COVID-19 Campus Lockdown: A Psychological Network Analysis
Source: Front Psychiatry. 2022 Jun 30;13:816298. doi: 10.3389/fpsyt.2022.816298 (PMC9280181; doi:10.3389/fpsyt.2022.816298)
Supplement: Supplementary file 1 [file Table_1.DOCX]

Supplementary Material

# Supplementary Tables

## Supplementary Table 1 Correlation matrix of Big Five Personality, K6 score, and six symptoms during college lockdown

| Variables | (1) | (2) | (3) | (4) | (5) | (6) | (7) | (8) | (9) | (10) | (11) | (12) |
| --- | --- | --- | --- | --- | --- | --- | --- | --- | --- | --- | --- | --- |
| (1) Extraversion | 1.000 |  |  |  |  |  |  |  |  |  |  |  |
|  |  |  |  |  |  |  |  |  |  |  |  |  |
| (2) Agreeableness | -0.029 | 1.000 |  |  |  |  |  |  |  |  |  |  |
|  | (0.514) |  |  |  |  |  |  |  |  |  |  |  |
| (3) Conscientiousness | 0.210 | 0.081 | 1.000 |  |  |  |  |  |  |  |  |  |
|  | (0.000) | (0.063) |  |  |  |  |  |  |  |  |  |  |
| (4) Neuroticism | -0.283 | -0.213 | -0.225 | 1.000 |  |  |  |  |  |  |  |  |
|  | (0.000) | (0.000) | (0.000) |  |  |  |  |  |  |  |  |  |
| (5) Openness | 0.130 | 0.004 | 0.074 | -0.067 | 1.000 |  |  |  |  |  |  |  |
|  | (0.003) | (0.936) | (0.092) | (0.128) |  |  |  |  |  |  |  |  |
| (6) K6 score | -0.099 | -0.053 | -0.127 | 0.280 | -0.017 | 1.000 |  |  |  |  |  |  |
|  | (0.023) | (0.224) | (0.004) | (0.000) | (0.694) |  |  |  |  |  |  |  |
| (7) Nervous | -0.080 | -0.038 | -0.033 | 0.255 | -0.075 | 0.786 | 1.000 |  |  |  |  |  |
|  | (0.068) | (0.382) | (0.444) | (0.000) | (0.085) | (0.000) |  |  |  |  |  |  |
| (8) Hopeless | 0.011 | -0.006 | -0.029 | 0.138 | 0.012 | 0.818 | 0.580 | 1.000 |  |  |  |  |
|  | (0.797) | (0.891) | (0.505) | (0.002) | (0.790) | (0.000) | (0.000) |  |  |  |  |  |
| (9) Restless | -0.100 | 0.012 | -0.109 | 0.254 | 0.024 | 0.847 | 0.666 | 0.627 | 1.000 |  |  |  |
|  | (0.022) | (0.782) | (0.012) | (0.000) | (0.589) | (0.000) | (0.000) | (0.000) |  |  |  |  |
| (10) Depress | -0.064 | -0.068 | -0.122 | 0.240 | -0.013 | 0.876 | 0.607 | 0.681 | 0.689 | 1.000 |  |  |
|  | (0.145) | (0.122) | (0.005) | (0.000) | (0.765) | (0.000) | (0.000) | (0.000) | (0.000) |  |  |  |
| (11) Effort | -0.107 | -0.099 | -0.150 | 0.266 | -0.034 | 0.848 | 0.598 | 0.634 | 0.614 | 0.743 | 1.000 |  |
|  | (0.014) | (0.023) | (0.001) | (0.000) | (0.430) | (0.000) | (0.000) | (0.000) | (0.000) | (0.000) |  |  |
| (12) Worthless | -0.136 | -0.064 | -0.168 | 0.228 | -0.001 | 0.805 | 0.483 | 0.607 | 0.598 | 0.646 | 0.637 | 1.000 |
|  | (0.002) | (0.146) | (0.000) | (0.000) | (0.979) | (0.000) | (0.000) | (0.000) | (0.000) | (0.000) | (0.000) |  |
|  | | | | | | | | | | | | |

## Supplementary Table 2 Correlation matrix of Big Five Personality, K6 score, and six symptoms after college lockdown

| Variables | (1) | (2) | (3) | (4) | (5) | (6) | (7) | (8) | (9) | (10) | (11) | (12) |
| --- | --- | --- | --- | --- | --- | --- | --- | --- | --- | --- | --- | --- |
| (1) Extraversion | 1.000 |  |  |  |  |  |  |  |  |  |  |  |
|  |  |  |  |  |  |  |  |  |  |  |  |  |
| (2) Agreeableness | -0.029 | 1.000 |  |  |  |  |  |  |  |  |  |  |
|  | (0.514) |  |  |  |  |  |  |  |  |  |  |  |
| (3) Conscientiousness | 0.210 | 0.081 | 1.000 |  |  |  |  |  |  |  |  |  |
|  | (0.000) | (0.063) |  |  |  |  |  |  |  |  |  |  |
| (4) Neuroticism | -0.283 | -0.213 | -0.225 | 1.000 |  |  |  |  |  |  |  |  |
|  | (0.000) | (0.000) | (0.000) |  |  |  |  |  |  |  |  |  |
| (5) Openness | 0.130 | 0.004 | 0.074 | -0.067 | 1.000 |  |  |  |  |  |  |  |
|  | (0.003) | (0.936) | (0.092) | (0.128) |  |  |  |  |  |  |  |  |
| (6) K6 score | -0.168 | -0.095 | -0.189 | 0.316 | -0.072 | 1.000 |  |  |  |  |  |  |
|  | (0.000) | (0.030) | (0.000) | (0.000) | (0.098) |  |  |  |  |  |  |  |
| (7) Nervous | -0.148 | -0.054 | -0.136 | 0.300 | -0.095 | 0.827 | 1.000 |  |  |  |  |  |
|  | (0.001) | (0.219) | (0.002) | (0.000) | (0.030) | (0.000) |  |  |  |  |  |  |
| (8) Hopeless | -0.090 | -0.043 | -0.108 | 0.219 | -0.032 | 0.845 | 0.623 | 1.000 |  |  |  |  |
|  | (0.040) | (0.325) | (0.013) | (0.000) | (0.467) | (0.000) | (0.000) |  |  |  |  |  |
| (9) Restless | -0.165 | -0.075 | -0.192 | 0.309 | -0.058 | 0.875 | 0.752 | 0.650 | 1.000 |  |  |  |
|  | (0.000) | (0.084) | (0.000) | (0.000) | (0.182) | (0.000) | (0.000) | (0.000) |  |  |  |  |
| (10) Depress | -0.126 | -0.130 | -0.150 | 0.275 | -0.023 | 0.901 | 0.643 | 0.723 | 0.749 | 1.000 |  |  |
|  | (0.004) | (0.003) | (0.001) | (0.000) | (0.592) | (0.000) | (0.000) | (0.000) | (0.000) |  |  |  |
| (11) Effort | -0.161 | -0.099 | -0.189 | 0.293 | -0.096 | 0.924 | 0.713 | 0.748 | 0.746 | 0.845 | 1.000 |  |
|  | (0.000) | (0.024) | (0.000) | (0.000) | (0.027) | (0.000) | (0.000) | (0.000) | (0.000) | (0.000) |  |  |
| (12) Worthless | -0.180 | -0.093 | -0.208 | 0.251 | -0.072 | 0.859 | 0.590 | 0.703 | 0.662 | 0.751 | 0.782 | 1.000 |
|  | (0.000) | (0.034) | (0.000) | (0.000) | (0.097) | (0.000) | (0.000) | (0.000) | (0.000) | (0.000) | (0.000) |  |
|  | | | | | | | | | | | | |

## Supplementary Table 3 Estimated edge weights of the psychological network during college lockdown (without control variables)

|  | Extraversion | Agreeableness | Conscientiousness | Neuroticism | Openness | Nervous | Hopeless | Restless | Depress | Effort | Worthless |
| --- | --- | --- | --- | --- | --- | --- | --- | --- | --- | --- | --- |
| Extraversion | 0 | 0 | 0.09 | -0.18 | 0.01 | 0 | 0 | 0 | 0 | 0 | 0 |
| Agreeableness | 0 | 0 | 0 | -0.15 | 0 | 0 | 0 | 0 | 0 | 0 | 0 |
| Conscientiousness | 0.09 | 0 | 0 | -0.10 | 0 | 0 | 0 | 0 | 0 | 0 | -0.03 |
| Neuroticism | -0.18 | -0.15 | -0.10 | 0 | 0 | 0.05 | 0 | 0.04 | 0 | 0.03 | 0 |
| Openness | 0.01 | 0 | 0 | 0 | 0 | 0 | 0 | 0 | 0 | 0 | 0 |
| Nervous | 0 | 0 | 0 | 0.05 | 0 | 0 | 0.11 | 0.31 | 0.06 | 0.14 | 0 |
| Hopeless | 0 | 0 | 0 | 0 | 0 | 0.11 | 0 | 0.12 | 0.22 | 0.10 | 0.15 |
| Restless | 0 | 0 | 0 | 0.04 | 0 | 0.31 | 0.12 | 0 | 0.24 | 0 | 0.13 |
| Depress | 0 | 0 | 0 | 0 | 0 | 0.06 | 0.22 | 0.24 | 0 | 0.37 | 0.15 |
| Effort | 0 | 0 | 0 | 0.03 | 0 | 0.14 | 0.10 | 0 | 0.37 | 0 | 0.19 |
| Worthless | 0 | 0 | -0.03 | 0 | 0 | 0 | 0.15 | 0.13 | 0.15 | 0.19 | 0 |

## Supplementary Table 4 Estimated edge weights of the psychological network after college lockdown (without control variables)

|  | Extraversion | Agreeableness | Conscientiousness | Neuroticism | Openness | Nervous | Hopeless | Restless | Depress | Effort | Worthless |
| --- | --- | --- | --- | --- | --- | --- | --- | --- | --- | --- | --- |
| Extraversion | 0 | -0.03 | 0.11 | -0.20 | 0.04 | 0 | 0 | 0 | 0 | 0 | -0.04 |
| Agreeableness | -0.03 | 0 | 0 | -0.12 | 0 | 0 | 0 | 0 | 0 | 0 | 0 |
| Conscientiousness | 0.11 | 0 | 0 | -0.10 | 0 | 0 | 0 | -0.01 | 0 | 0 | -0.05 |
| Neuroticism | -0.20 | -0.12 | -0.10 | 0 | 0 | 0.05 | 0 | 0.05 | 0 | 0.02 | 0 |
| Openness | 0.04 | 0 | 0 | 0 | 0 | 0 | 0 | 0 | 0 | 0 | 0 |
| Nervous | 0 | 0 | 0 | 0.05 | 0 | 0 | 0.09 | 0.39 | 0 | 0.21 | 0 |
| Hopeless | 0 | 0 | 0 | 0 | 0 | 0.09 | 0 | 0.02 | 0.15 | 0.19 | 0.19 |
| Restless | 0 | 0 | -0.01 | 0.05 | 0 | 0.39 | 0.02 | 0 | 0.24 | 0.08 | 0.04 |
| Depress | 0 | 0 | 0 | 0 | 0 | 0 | 0.15 | 0.24 | 0 | 0.44 | 0.17 |
| Effort | 0 | 0 | 0 | 0.02 | 0 | 0.21 | 0.19 | 0.08 | 0.44 | 0 | 0.29 |
| Worthless | -0.04 | 0 | -0.05 | 0 | 0 | 0 | 0.19 | 0.04 | 0.17 | 0.29 | 0 |

## Supplementary Table 5 Estimated edge weights of the psychological network during college lockdown (with control variables)

|  | Female | Age | Grade 2 | Grade 3 | Grade 4 | Extraversion | Agreeableness | Conscientiousness | Neuroticism | Openness | Nervous | Hopeless | Restless | Depress | Effort | Worthless |
| --- | --- | --- | --- | --- | --- | --- | --- | --- | --- | --- | --- | --- | --- | --- | --- | --- |
| Female | 0 | 0 | 0 | 0 | 0 | 0 | 0 | 0 | 0.05 | 0 | 0 | 0 | 0 | 0 | 0 | 0 |
| Age | 0 | 0 | 0.71 | 1.44 | 1.73 | 0 | 0 | 0 | 0 | 0 | 0 | 0 | 0 | 0 | 0 | 0 |
| Grade 2 | 0 | 0.71 | 0 | -3.24 | -2.74 | 0 | 0 | 0 | 0 | 0 | 0 | 0 | 0 | 0 | 0 | 0 |
| Grade 3 | 0 | 1.44 | -3.24 | 0 | -3.91 | 0 | 0 | 0 | 0 | 0 | 0 | 0 | 0 | 0 | 0 | 0 |
| Grade 4 | 0 | 1.73 | -2.74 | -3.91 | 0 | 0 | 0 | 0 | 0 | 0 | 0 | 0 | 0 | 0 | 0 | 0 |
| Extraversion | 0 | 0 | 0 | 0 | 0 | 0 | 0 | 0.09 | -0.18 | 0.01 | 0 | 0 | 0 | 0 | 0 | 0 |
| Agreeableness | 0 | 0 | 0 | 0 | 0 | 0 | 0 | 0 | -0.15 | 0 | 0 | 0 | 0 | 0 | 0 | 0 |
| Conscientiousness | 0 | 0 | 0 | 0 | 0 | 0.09 | 0 | 0 | -0.10 | 0 | 0 | 0 | 0 | 0 | 0 | -0.03 |
| Neuroticism | 0.05 | 0 | 0 | 0 | 0 | -0.18 | -0.15 | -0.10 | 0 | 0 | 0.05 | 0 | 0.03 | 0 | 0.03 | 0 |
| Openness | 0 | 0 | 0 | 0 | 0 | 0.01 | 0 | 0 | 0 | 0 | 0 | 0 | 0 | 0 | 0 | 0 |
| Nervous | 0 | 0 | 0 | 0 | 0 | 0 | 0 | 0 | 0.05 | 0 | 0 | 0.11 | 0.31 | 0.06 | 0.14 | 0 |
| Hopeless | 0 | 0 | 0 | 0 | 0 | 0 | 0 | 0 | 0 | 0 | 0.11 | 0 | 0.12 | 0.22 | 0.10 | 0.15 |
| Restless | 0 | 0 | 0 | 0 | 0 | 0 | 0 | 0 | 0.03 | 0 | 0.31 | 0.12 | 0 | 0.24 | 0 | 0.13 |
| Depress | 0 | 0 | 0 | 0 | 0 | 0 | 0 | 0 | 0 | 0 | 0.06 | 0.22 | 0.24 | 0 | 0.37 | 0.15 |
| Effort | 0 | 0 | 0 | 0 | 0 | 0 | 0 | 0 | 0.03 | 0 | 0.14 | 0.10 | 0 | 0.37 | 0 | 0.19 |
| Worthless | 0 | 0 | 0 | 0 | 0 | 0 | 0 | -0.03 | 0 | 0 | 0 | 0.15 | 0.13 | 0.15 | 0.19 | 0 |

## Supplementary Table 6 Estimated edge weights of the psychological network after college lockdown (with control variables)

|  | Female | Age | Grade 2 | Grade 3 | Grade 4 | Extraversion | Agreeableness | Conscientiousness | Neuroticism | Openness | Nervous | Hopeless | Restless | Depress | Effort | Worthless |
| --- | --- | --- | --- | --- | --- | --- | --- | --- | --- | --- | --- | --- | --- | --- | --- | --- |
| Female | 0 | 0 | 0 | 0 | 0 | 0 | 0 | 0 | 0.05 | 0 | 0 | 0 | 0 | 0 | 0 | 0 |
| Age | 0 | 0 | 0.48 | 1.18 | 1.60 | 0 | 0 | 0 | 0 | 0 | 0 | 0 | 0 | 0 | 0 | 0 |
| Grade 2 | 0 | 0.48 | 0 | -2.65 | -2.51 | 0 | 0 | 0 | 0 | 0 | 0 | 0 | 0 | 0 | 0 | 0 |
| Grade 3 | 0 | 1.18 | -2.65 | 0 | -3.68 | 0 | 0 | 0 | 0 | 0 | 0 | 0 | 0 | 0 | 0 | 0 |
| Grade 4 | 0 | 1.60 | -2.51 | -3.68 | 0 | 0 | 0 | 0 | 0 | 0 | 0 | 0 | 0 | 0 | 0 | 0 |
| Extraversion | 0 | 0 | 0 | 0 | 0 | 0 | 0 | 0.08 | -0.18 | 0.02 | 0 | 0 | 0 | 0 | 0 | -0.02 |
| Agreeableness | 0 | 0 | 0 | 0 | 0 | 0 | 0 | 0 | -0.13 | 0 | 0 | 0 | 0 | 0 | 0 | 0 |
| Conscientiousness | 0 | 0 | 0 | 0 | 0 | 0.08 | 0 | 0 | -0.09 | 0 | 0 | 0 | -0.01 | 0 | 0 | -0.04 |
| Neuroticism | 0.05 | 0 | 0 | 0 | 0 | -0.18 | -0.13 | -0.09 | 0 | 0 | 0.05 | 0 | 0.04 | 0 | 0.02 | 0 |
| Openness | 0 | 0 | 0 | 0 | 0 | 0.02 | 0 | 0 | 0 | 0 | 0 | 0 | 0 | 0 | 0 | 0 |
| Nervous | 0 | 0 | 0 | 0 | 0 | 0 | 0 | 0 | 0.05 | 0 | 0 | 0.09 | 0.39 | 0 | 0.21 | 0 |
| Hopeless | 0 | 0 | 0 | 0 | 0 | 0 | 0 | 0 | 0 | 0 | 0.09 | 0 | 0.02 | 0.15 | 0.19 | 0.19 |
| Restless | 0 | 0 | 0 | 0 | 0 | 0 | 0 | -0.01 | 0.04 | 0 | 0.39 | 0.02 | 0 | 0.24 | 0.08 | 0.04 |
| Depress | 0 | 0 | 0 | 0 | 0 | 0 | 0 | 0 | 0 | 0 | 0 | 0.15 | 0.24 | 0 | 0.44 | 0.17 |
| Effort | 0 | 0 | 0 | 0 | 0 | 0 | 0 | 0 | 0.02 | 0 | 0.21 | 0.19 | 0.08 | 0.44 | 0 | 0.29 |
| Worthless | 0 | 0 | 0 | 0 | 0 | -0.02 | 0 | -0.04 | 0 | 0 | 0 | 0.19 | 0.04 | 0.17 | 0.29 | 0 |

# Supplementary Figures


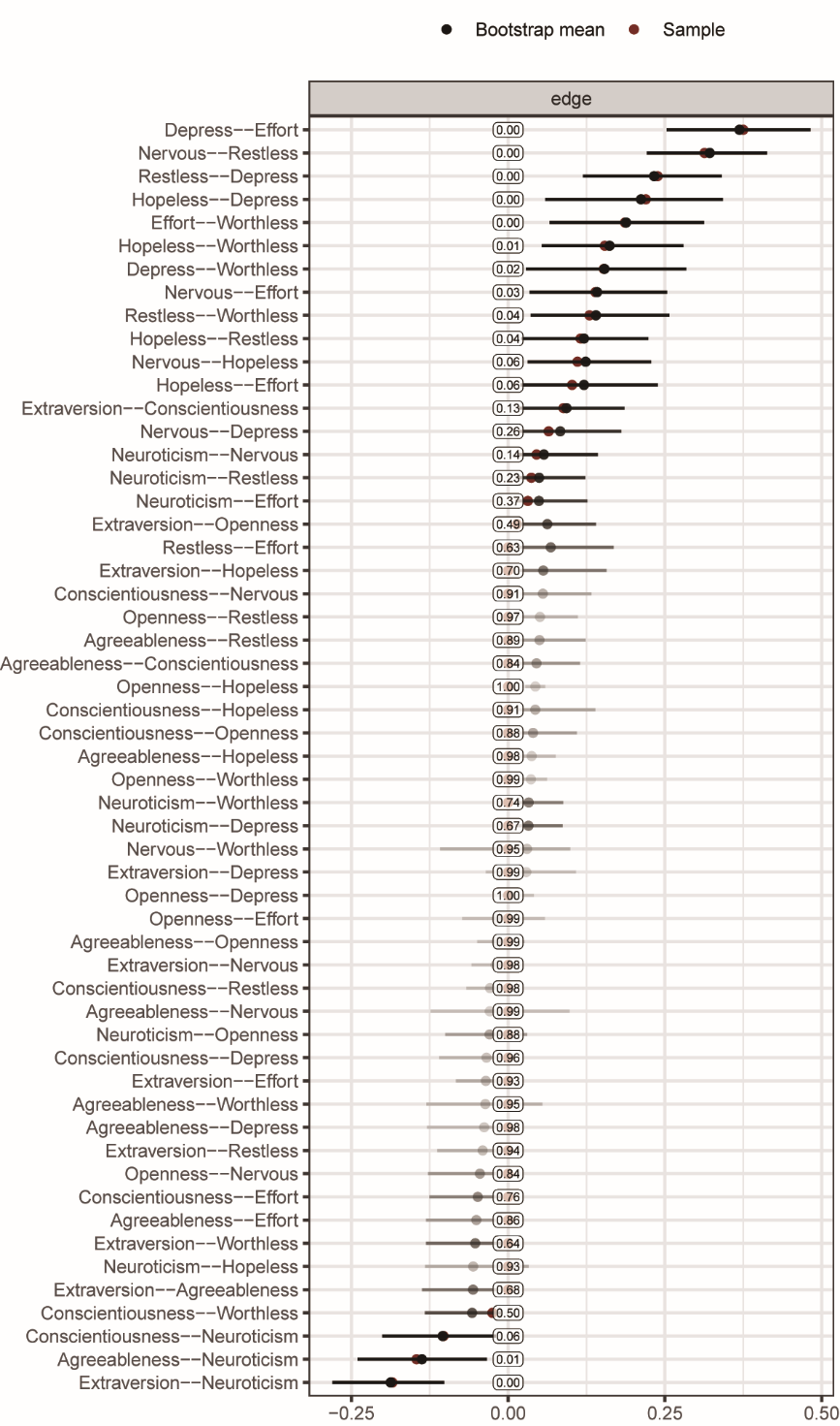


## Supplementary Figure 1 Accuracy of edge weights during lockdown without control variables. The x-axle indicates the edge weights and the y-axle indicates the nodes linked by the edges. The black dots denote the mean value of the bootstrapped edge weights and the red dots denote the edge weights from current sample. The black lines denote the 95% confidence intervals of the bootstrapped sample.


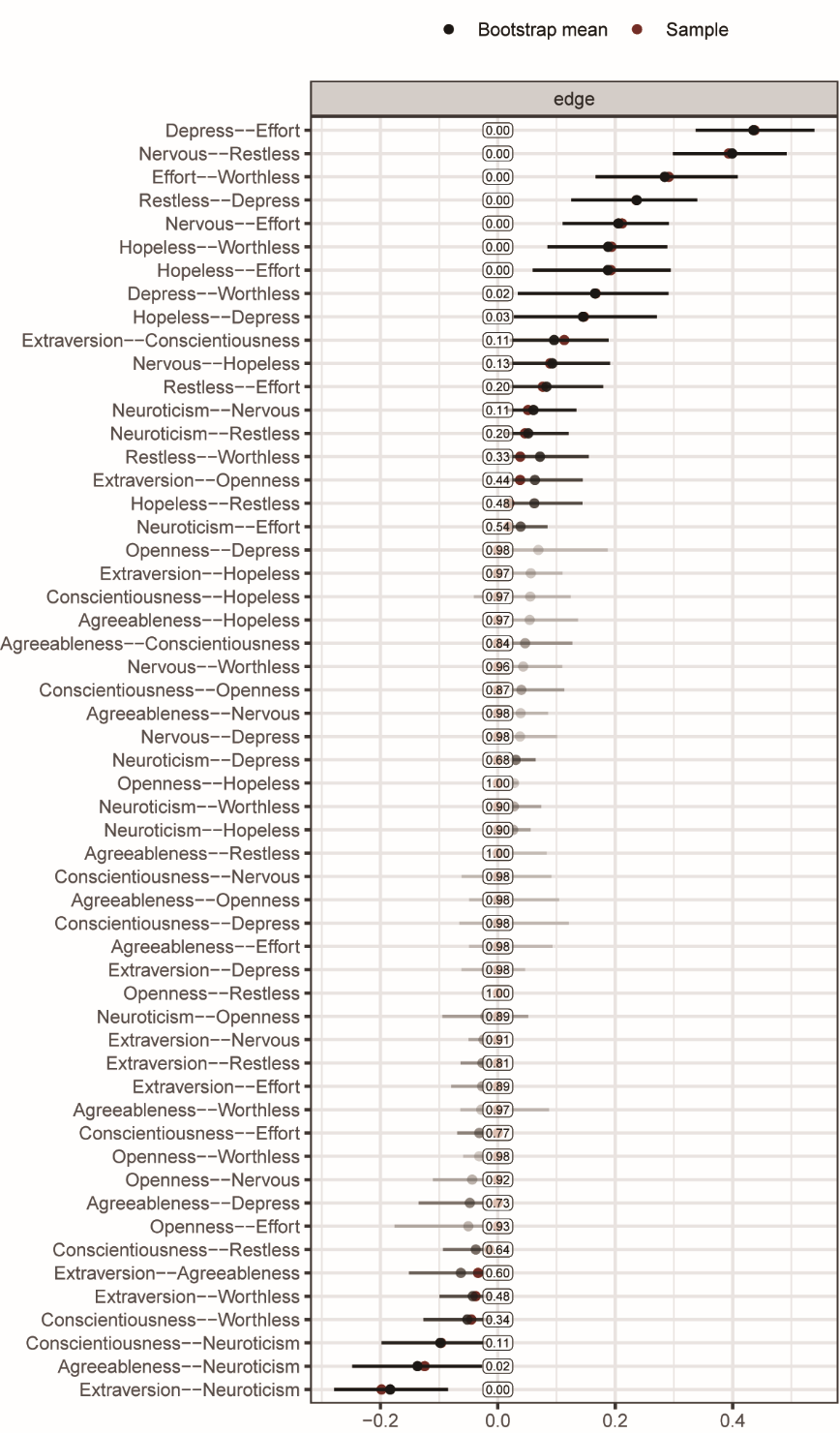


## Supplementary Figure 2 Accuracy of edge weights after lockdown without control variables. The x-axle indicates the edge weights and the y-axle indicates the nodes linked by the edges. The black dots denote the mean value of the bootstrapped edge weights and the red dots denote the edge weights from current sample. The black lines denote the 95% confidence intervals of the bootstrapped sample.


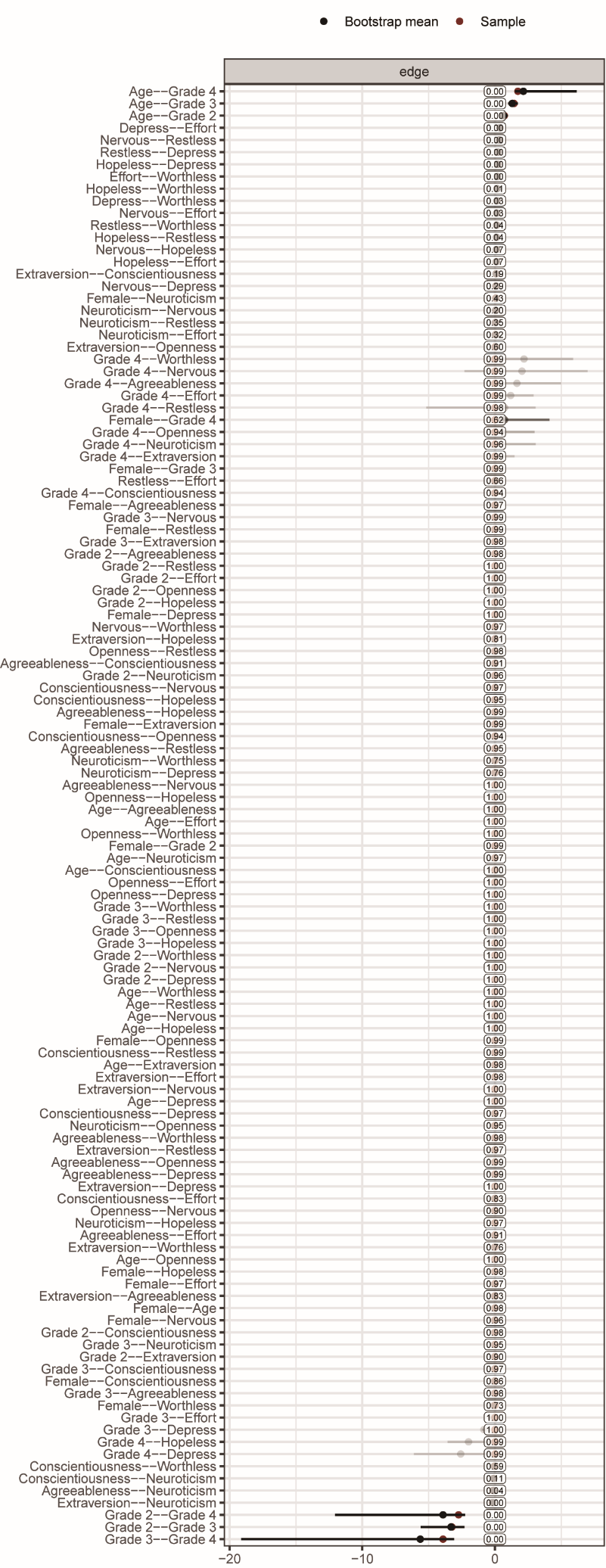


## Supplementary Figure 3 Accuracy of edge weights during lockdown with control variables. The x-axle indicates the edge weights and the y-axle indicates the nodes linked by the edges. The black dots denote the mean value of the bootstrapped edge weights and the red dots denote the edge weights from current sample. The black lines denote the 95% confidence intervals of the bootstrapped sample.


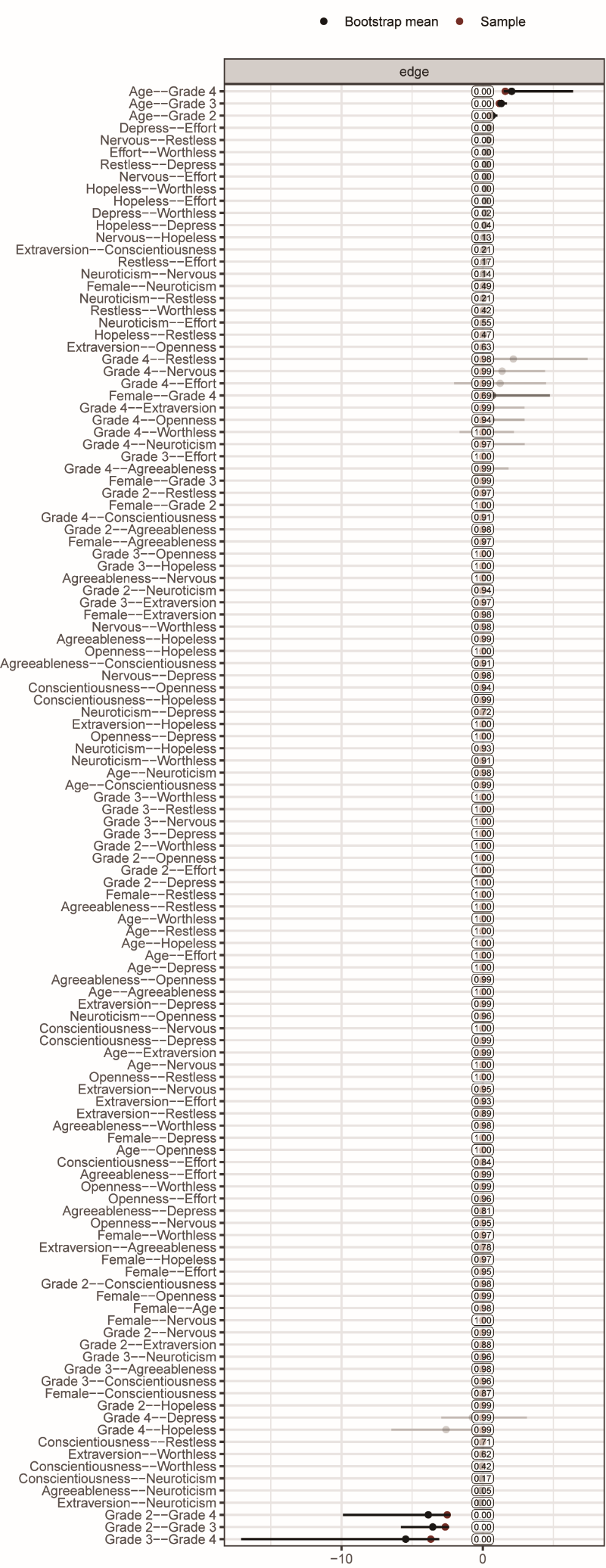


## Supplementary Figure 4 Accuracy of edge weights after lockdown with control variables. The x-axle indicates the edge weights and the y-axle indicates the nodes linked by the edges. The black dots denote the mean value of the bootstrapped edge weights and the red dots denote the edge weights from current sample. The black lines denote the 95% confidence intervals of the bootstrapped sample.


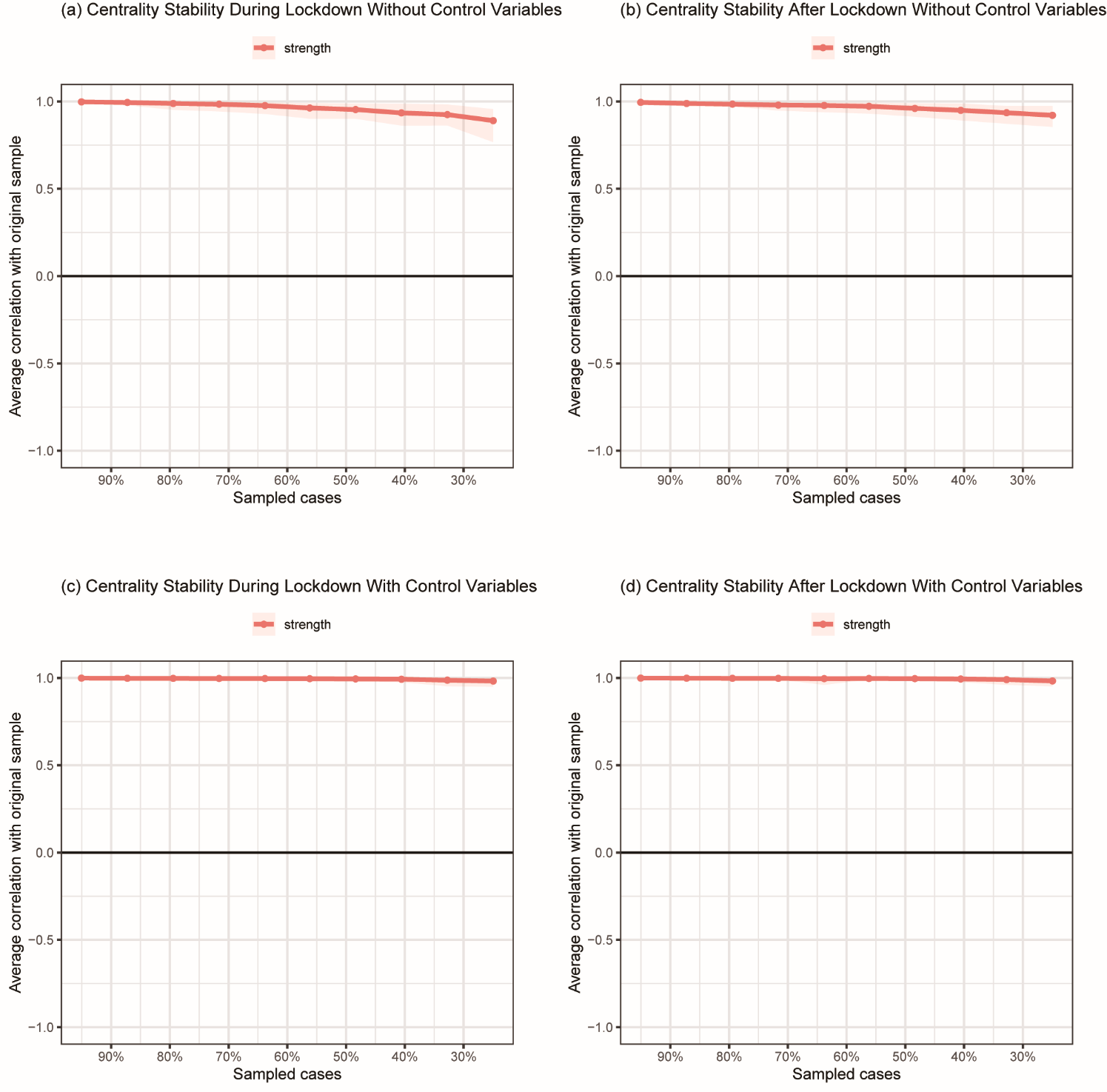


## Supplementary Figure 5 Centrality stability results.
